# Coping Mechanisms and Therapy for Children with Trauma-Induced Auditory Hallucinations

**DOI:** 10.1192/j.eurpsy.2026.10573

**Published:** 2026-07-31

**Authors:** M. A. K. Guirguis, H. Raai

**Affiliations:** Psychiatry, SBH Health System, New York, United States

## Abstract

**Introduction:**

Auditory hallucinations are a debilitating manifestation of trauma in children. Voice-hearing is common among youth exposed to multiple traumatic experiences and is associated with cognitive mechanisms that contribute to the persistence of symptoms [1]. These experiences can cause severe distress, disrupt development, and may increase risk of self-harm mediated by hallucinatory symptoms [5]. Children often attempt to manage these experiences through coping strategies such as humming, listening to music, engaging in play, or externalizing the voices into objects or characters—techniques aligned with distraction, self-generated competing stimuli, and externalization described in coping-strategy-enhancement frameworks [2]. While antipsychotic medications remain an option in severe cases [3], creative coping strategies can be integrated into structured trauma-focused therapies [2,4].

**Objectives:**

This study explores coping mechanisms for trauma-induced hallucinations in children, with emphasis on idiosyncratic strategies such as puppet play, and evaluates psychiatric treatment management including psychopharmacology and trauma-focused psychotherapies.

**Methods:**

A clinical and literature review was conducted, drawing on Thomas et al (2014), Adetunji et al (2005), and Reid et al (2024). Findings were contextualized through a case illustration of a 9-year-old girl with trauma-related hallucinations who used puppets to act out and externalize frightening voices.

**Results:**

The patient’s puppet play reflected key CSE principles—distraction, externalization, and normalization—demonstrating how spontaneous coping aligns with structured interventions [2]. Pharmacological evidence supports risperidone when hallucinations remain severe [3]; however, in this case, medication was reserved given her adaptive strategy. Trauma-focused therapies such as TF-CBT and EMDR could incorporate puppet play to facilitate safe narrative reprocessing and emotional regulation [1,4].

**Image 1:**
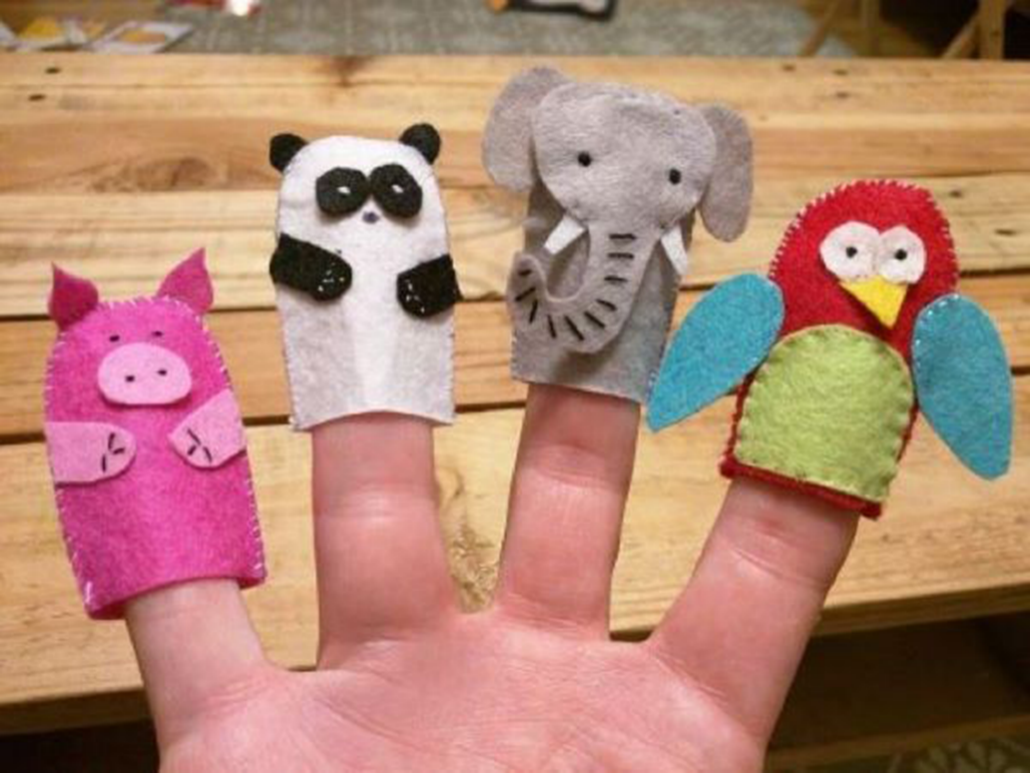

**Conclusions:**

Children may naturally generate innovative coping strategies for trauma-induced hallucinations. Clinicians should recognize and support these behaviors within a multimodal treatment framework that integrates psychosocial techniques, judicious pharmacotherapy, and trauma-focused psychotherapies.

**Disclosure of Interest:**

None Declared

